# Control of malaria in the Comoro Islands over the past century

**DOI:** 10.1186/s12936-017-2027-1

**Published:** 2017-09-26

**Authors:** Ismaël Chakir, Ali Ibrahim Said, Bacar Affane, Ronan Jambou

**Affiliations:** 1 Unité d’Immunologie, Institut Pasteur de Madagascar, Antananarivo, Madagascar; 2 Département de Biochimie, Université des Comores/Centre Universitaire de Patsy, Patsy, Comoros; 3 Programme National de Lutte contre le Paludisme (PNLP), Ministère de la Santé des Comores, Moroni, Comoros; 4 Département Parasitologie et Insectes Vecteurs, Institut Pasteur, Paris, France; 5 Present Address: Département de Parasitologie et Mycologie, Institut Pasteur de Côte d’Ivoire, BP 490, Abidjan, 01 Ivory Coast

**Keywords:** Comoro Islands, Malaria, Artemisinin, Chloroquine, Anopheles

## Abstract

**Background:**

The Comoros are an archipelago located in the Indian Ocean between the eastern coasts of Africa and north of Madagascar. Malaria transmission appeared late in the 19th century due to the intensification of human migration. The story of malaria transmission for the past century is depicted to provide useful lessons for the future. Currently, malaria transmission occurs differently on each island; thus, control strategies must be adapted for each particular island. Tentative malaria control in Comoros has a long history of success and failure. This study reviews the data available as a basis for recommendations for the future.

**Results:**

There has been much effort to reach a pre-eradication state in Anjouan and Moheli, but only control steps have been taken in the Great Comoro. To date, the primary strategy used is mass treatment of the population using artemisinin-based combination therapy (ACT), which is similar to the strategy deployed during the 1950s in other countries. ACT appears efficient in two of the three islands; however, the sustainability of the strategy is unknown. This sustainability is compromised by (i) the huge level of uncontrolled exchange between the Comoro Islands and their neighbours, increasing the risk of introducing ACT-resistant strains, (ii) the use of large quantities of pesticides for agriculture usually associated with the resistance of mosquitoes, and (iii) the cost of the actions themselves.

**Conclusions:**

In view of the history of malaria in this area, the first recommendation is to enhance the training of health workers and the population. The second step is to establish a national strategy to assess malaria and related factors, which is currently lacking. A survey to assess the drug sensitivity of the parasites is particularly important in a context of low transmission associated with mass treatment of the population. The last point should be to secure financial support, which is not obvious in a context of pre-elimination. The Comoro Islands are thus a living laboratory to experiments with strategies for elimination, but the future is complex.

## Background

The Comoros are an archipelago located in the Indian Ocean in the northern part of the Mozambique Channel, between the eastern coast of Africa and north of Madagascar. It is composed of four volcanic islands: the Great Comoro (Ngazidza), Moheli (Mwali), Anjouan (Ndzuwani), and Mayotte (Maoré) and a series of unoccupied reefs. The Union of the Comoros is composed of only three islands, as Mayotte decided to remain French when the other islands became independent in 1975. The population was reported to be 652,202 inhabitants in 2008 in an area of 2236 km^2^. According to the World Health Organization (WHO), the country is ranked 134th out of 177 for the Human Development Index. Moreover, 44.8% of the inhabitants live under the poverty threshold. Due to its volcanism, its human settlement, its culture and ecology, this archipelago has a unique epidemiological biotope [1–3].

In this part of the world, malaria has a relatively recent history following human settlement and migration. Malaria did not exist in the country in the 17th century, but European travellers reported intermittent fever after the middle of the eighteenth century [1]. Malaria is both a major public health issue and a socio-economic problem threatening each family due to the cost of treatment. In the paediatric ward of the regional hospital of Moroni, between 36 and 46% of children and more than 50% of babies attend the hospital for malaria. However, due to the diversity of the ecological biotope, malaria transmission differs significantly from one island to another and even more between two hillsides of the same island [4]. Due to an intense emigration of the Comorian community, many imported malaria cases have spread to other countries, such as France. In Moheli, the two main vectors are *Anopheles gambiae* s.s. and *Anopheles funestus*. These mosquitoes are frequently found on the southern coast, where the estuaries represent the major larval breeding sites during the dry season [1, 3, 5]. However, their density depends on the rains and, for *An. funestus*, on the blockage of estuaries during the dry season. *Anopheles gambiae* s.s. is the only vector on the Northern and Eastern coasts. *Anopheles gambiae* s.s. and *An. funestus* both proliferate on the Southwest coast, where malaria is highly endemic. In the two plateaux of the central and southern massifs, only *An. gambiae* s.s. is found [1, 3, 5]. Anjouan has a larger variety of epidemiology related to its tropical ecology. The Great Comoros is characterized by a virtual shortage of surface water and by artificial anthropic reservoirs (cisterns and ablution basins) [6].

Despite active malaria control in Mayotte for the past two decades, recent studies highlight the presence of *Anopheles merus* and *An. gambiae* [7]. Since 2001, indoor house spreading (IHS) of deltamethrin has been widely used, and impregnated bed nets are freely distributed to pregnant women and newborn children.

Facing the diversity in malaria vectors and parasites, control actions must be adapted locally. However, the tentative control of malaria has a long history of success and failure [8]. The last episode of this battle started in June 2007 with a strategic plan focusing on (i) reinforcement of public health structures, (ii) improvement of sanitation and (iii) distribution of long-lasting impregnated bed nets (LLIN) over the three islands, but targeting pregnant women and children younger than 5 years of age. Since 2007, this plan was completed in Moheli by indoor house spraying with insecticides and mass drug administration (MDA) of artemether–piperaquine [1]. This combination is thought to decrease the risk of drug resistance and to be active against *Plasmodium vivax*. This intensive strategy resulted in a drastic change in the number of cases [1]. MDA supported by Chinese partners gave encouraging results with a clear improvement of indicators in Moheli and Anjouan. At the end of 2013, the same programme started in the Great Comoro involving mass treatment.

However, the future of these efforts deployed by the authorities and supported by international partners remains unclear [9]. The topic of this review was to summarize different phases of the tentative control of malaria in Comoros by the government, stakeholders and partners over the past century in the Comoro Islands. The results of these actions provide valuable information to discuss a new plan for elimination.

## The Comoro Islands

The Comoros archipelago is located north of Madagascar, between 11°20′ and 13°04′ Southern latitude and between 42° and 45° eastern longitude. Moheli (374 km^2^) is quite flat with a slim coastal plain, where the estuaries of streams are blocked during the dry season by an offshore bar. In the centre, a plateau located at an altitude of 100 and 300 m has a permeable soil that allows the formation of lakes and ponds. The tropical climate varies according to the altitude and exposure to wind. Hillsides exposed to the wind experience heavy rainfalls whereas non-exposed hillsides remain dry. There are two seasons: a hot, rainy season from November to April and a dry, cold season from May to October. On the coast, the annual average temperature is above 23 °C with 2 m of rain annually. At altitude in the centre of the island, the weather is colder but with more rainfall.

Anjouan has an area of 424 km^2^. The M’tingui Mountain peaks at an altitude of 1578 m, from which permanent streams flow, creating estuaries. They are also obstructed during the dry season, which creates polluted lagoons. The impermeability of the soil causes floods and creates swamps during the wet season. The coastal plains are rare and narrow, with population localized in two fertile plateaux located between 300 and 500 m altitude [3].

The Great Comoro is the largest island (1024 km^2^), and it has the highest peak (2361 m at the top of the active volcano Karthala). This volcano began to emerge in the Quaternary period and is still rising. Despite 3 m of rain annually, due to its recent un-decomposed and permeable volcanic soil and its very sharp slopes, there is no permanent stream or damp areas. Populations store water in large domestic cisterns, which are the main breeding sites for mosquitoes.

The settlement of the Comoros began with Arabian migrants coming from the Gulf via Zanzibar. They brought African slaves with them (locally called ‘Mozambicans’). They established an independent sultanate on each island. Later, in the 18th century, Malagasy pirates settled in Mayotte and Moheli, where they kept their mother tongue, while Swahili remained the most common spoken language [1–3]. Most of the invaders came from Madagascar with the objective to steal but also to settle. Mayotte was bought by France in 1841. During the 19th century, conflicts occurred between the different sultans of the archipelagos, which caused them to ask for French protection. This relationship paved the way for colonization, which began as early as 1843 but was officially established on July 25th, 1912. Colonization ended on July 6th, 1975, but the population of Mayotte chose to remain French.

**Fig. 1:**
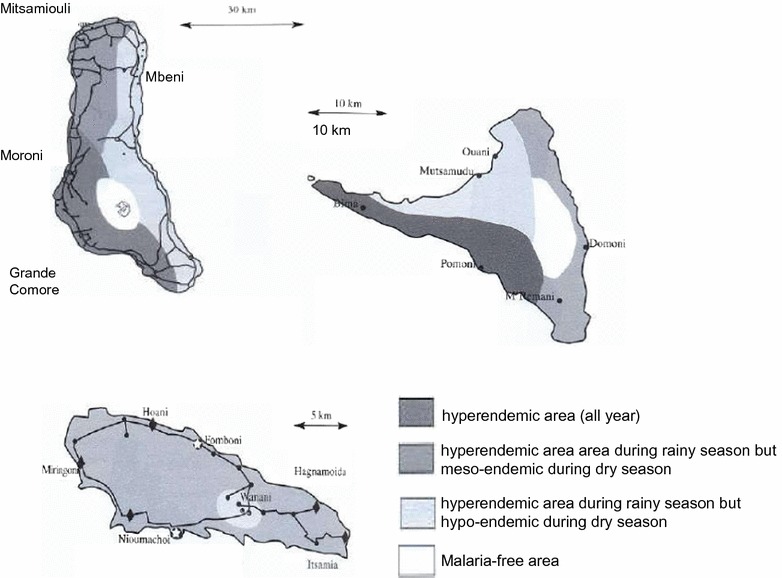
Transmission of malaria in the Comoro Islands. Representation of the different areas of malaria transmission of the three islands of the Islamic Republic of Comoro (Mayotte is not included). The grey scale is related to the type of transmission

The geographic and climatic diversity of the islands clearly affects the diversity of the mosquito populations [3]. Following these ecological conditions, two main epidemiological profiles can be described in Comoros for malaria transmission (Fig. 1): (i) areas of permanent transmission, including all parts of the Great Comoro due to the cisterns and their overflow, the Western and Eastern coast of Anjouan with its narrow plains and all the coast of Moheli [10], and (ii) areas of meso-endemic transmission, including regions of altitude in Anjouan and Moheli. Mayotte undergoes the same situation with variations during the rainy season. The repartition of both *An. funestus* and *An. gambiae* depends on rain and humidity during the wet season [1, 3, 5].

## History of malaria in the Comoro Islands

### The early description

In the Indian Ocean, malaria spread via human migration and the transportation of mosquitoes by hurricanes and boats. Most data from this period were obtained through administrative reports written by crews of the boats. A high level of mortality by fever slowed down the colonization of Madagascar [11, 12], and in 1643, one-third of the “le Saint-Laurent” boat crew died from fever [13]. At the end of the 19th century, a dramatic malaria epidemic occurred in Mauritius and Réunion [14], followed by Mayotte [15]. However, the real period of the introduction of malaria in each Comoro Island is not well established, except for the Great Comoro [3]. According to White and Bowen, Mayotte was renowned as a healthy place without fever until 1703. However, from 1792 to the beginning of the 19th century, intensive slave trade occurred between the African continent, Mayotte and Bourbon Island, and successive invasions of Malagasy people occurred. *Plasmodium falciparum* and its vector *An. gambiae* likely settled in the island during this period. Indeed, Lebeau, a surgeon working in Mayotte from 1840 to 1848, described new epidemics of discontinuous fevers experienced by a large portion of the inhabitants of the island. Daulle confirmed this description between 1852 and 1857. The epidemic, death rate and morbidity related to malaria among Mayotte’s population was exhaustively described by Blin from the colonial administration [16]. In 1918, the splenic index in schools ranged between 80 and 100% [10, 16, 17]. Among navigators, the insalubrity of Mayotte and Moheli was usually opposed to the relative healthiness of the anchorage to the north of Anjouan and to the absence of fever in the Great Comoro until 1920 [2].

In Moheli, malaria (intermittent fever) was present as early as the 15th century **[** 3]. During the 18th century, the quality of the anchorage of Anjouan was improved, but malaria was introduced by the middle of the 19th century [18, 19] before spreading to the centre of the island at the beginning of the 20th century. Similarly, the Great Comoro was considered a healthy place without anopheles due to the lack of permanent water until a first deadly epidemic of malaria occurred in 1920. An extension of malaria transmission throughout the island was registered during the wet season of 1923 [20]. From 1922 to 1924, epidemics occurred in all areas under 500 m of altitude with a very high mortality rate among the immunologically naïve population. A quarter of the population died during this time. The beginning of the epidemic was related to the building of water cisterns, where *An. gambiae* bred [21]. Supported by the economic expansion following the First World War, associated with the trade of vanilla, cisterns were built by the local population. Malaria transmission became rapidly hyperendemic in the island [2, 3]. In 1925, its spreading was confirmed throughout the island, particularly in the South Western and Western coasts [1]. During this period, malaria epidemics also occurred in Mauritius (1866–1867) and La Réunion (1868) [22].

### The description of the Anopheles species in the Comoros

The *Culicidae* fauna in Comoros remained virtually unknown until the end of the Second World War. However, the high mortality due to malaria and the occurrence of lymphatic filariasis led researchers to conduct several studies. In 1941, Lavergue (cited by [5]) described the presence of four *Anopheles* species and one *Aedes* species in the archipelago. A few years later, studies on Bancroft filariasis in Moheli, Anjouan, and Mayotte [23, 24] led to the description of seven new species: *Anopheles coustani, An. funestus, An. gambiae, Anopheles maculipalpis, Anopheles mascarensis* and *Anopheles pretoriensis* [21]. This settlement of *Anopheles* appears to be recent. The *An. mascarensis* species originates from Madagascar whereas the other five species are common all over the African continent. Blanchy also suspected the present of *An. merus* and of *Anopheles arabiensis* [3, 5, 21, 25], which were imported into the archipelago from the East African coast. Cytogenetic studies also suggested that *An. gambiae* s.s. [26] could have been transported from Africa during hurricanes [21]. Likewise, *An. funestus* was likely transported from Africa because the population found in Moheli is genetically distinct from the Malagasy population.

**Fig. 2:**
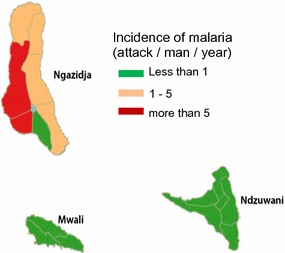
Malaria transmission in Comoros after mass treatment in 2014. Representation of the malaria transmission of the three islands of the Islamic Republic of Comoro (Mayotte is not included) after mass treatment with artemisinin-containing therapy

### Early attempts at control

Facing this appearance of malaria, the archipelago colonial administration attempted several control strategies. In Anjouan, at the beginning of the 20th century, channels were dug in the valleys of Bandacoua and Chicony to drain water. In 1924–1925 in the Great Comoro, a systematic mass drug administration of quinine was introduced to block an epidemic of malaria that caused more than 1000 deaths [20]. Stocks of quinine were provided for each village and given for free. Simultaneously, coconut oil was spread on puddles, and cisterns were closed and controlled.

Studies were intensified and conducted by the colonial Health Services of Madagascar and Dependences, in cooperation with ORSTOM (a French Institute for research overseas). In 1949, the parasitological index in patients at the hospital varied between 10 and 30% and in rural areas between 69 and 83%. They confirmed that *P. falciparum* was the only *Plasmodium* transmitted on the islands.

Between 1945 and 1957, the first large campaign targeting the elimination of malaria was organized in Mayotte and Moheli using quarterly indoor house spraying (HIS) of DDT and dieldrin. In 1956, these actions were extended to Anjouan, and in 1957 to the Great Comoro. In 1972, preliminary studies were also conducted in Moroni [27]. However, due campaigns against DTT, experimentation with malathion was performed in Moheli but were not extended due to the smell of the product [28].

In Mayotte, quarterly fenitrothion in-house sprayings were very efficient and almost reached the goal of eradicating malaria [6]. They were extended in 1972 to Anjouan and Mohéli with the association of DDT–fenitrothion. From 1973 to 1974, temephos was sprayed to fight against mosquito larvae in Mayotte and in the Great Comoro [8, 17, 29, 30]. These actions were only conducted for a short time and were poorly evaluated. Few data are available on the effectiveness of these sprayings, but in 1972 and 1976, the plasmodic index was still 36.5 and 25.5%, respectively [16], in these two islands. The side effect of these programmes was the first description in 1967 of DDT-resistant *Culex quinquefasciatus* by Chauvet, rapidly followed by resistance to dieldrin and propoxur (1987), which can be the first sign of the selection of mutated insects under treatment pressure. The presence of *Culex quinquefasciatus* and *Aedes aegypti* is associated with the transmission of *Wuchereria bancrofti* and of yellow fever throughout the archipelago [31], but the nuisance induced by these insects is helpful to promote the use of bed nets and curtains by the inhabitants [32].

After independence, activities to control the vectors slowed down from 1975 to 1986. This was due to the cost of control programmes facing financial difficulties of the new independent administration and to political instability. Simultaneously, resistance to chloroquine was first reported in 1980 and confirmed during a systematic survey in 1984 **[** 33, 34]. Chloroquine resistance was confirmed in vitro in 1987 [35]. By 2001, 29% of the isolates were resistant to chloroquine, and resistance to sulfadoxine/pyrimethamine reached 14%, leading to a review of the national treatment strategy [36, 37].

## A National Malaria Control Programme (NMCP) in Comoros

In agreement with the international commitments signed by the Comorian State, a plan was launched to fight against malaria in 1978 [17]. This national programme was funded by a cooperative initiative between all the stakeholders working at different levels to improve public health [37, 38], with the financial support of the WHO, United Nation Development Programme (UNDP) and UNICEF. Many training activities were conducted at all levels of the health system (malariologists, medical entomologists, technicians, and microscopists). Approximately 400 nurses, midwives and doctors were trained for the diagnosis, treatment and prevention of malaria. In 1989, dispensaries were equipped with microscopes and confirmation of the cases was thus possible, as was the organization of in vivo drug resistance assays. These studies highlighted a rapid increase of chloroquine-resistant parasites.

In 1987, activities for the control of malaria vectors resumed with the organization of the first national programme for the control of malaria led by the government since independence [29]. Both insecticides and fishes (*Poecilia reticulata*) were used. Indeed, in the archipelago, there are 1365 mosques and 1079 in the Great Comoros alone. Each mosque has a basin, which is an efficient breeding site for mosquitos [39]. The use of fishes in these basins was proposed in accordance with a very old tradition. This strategy was supported by the government, which facilitated the production of *Poecilia reticulata* after a preliminary trial in La Réunion [40]. Despite a very weak efficacy of this strategy [36], it is still used in Ngazidja, Ndzouani and Mwali. Téméphos was also proposed to treat these basins, but the quantity of product must be calculated for each basin, making it complicated to apply [36–38]. Simultaneously, indoor residual spraying (IRS) of malathion was implemented in Anjouan and Moheli, as was the distribution of impregnated bed nets and curtains in Anjouan, Moheli and the Great Comoro. These actions lasted for 3 years with a significant reduction of the vector density and the infection rate [29], but they were rapidly interrupted due to a lack of funds and inefficient organization [38]. Deltamethrin, lambda-cyhalothrin, and cyfluthrin are still used, but they require quarterly application [38].

These actions became better organized during the 1992–1997 period, and in 1992, a first guide on case management was published for health workers. Impregnated mosquito nets were distributed in Domoni (Moheli), Jéjé (Anjouan) and Djoumoichongo-Komioni (the Great Comoro) and sold by UNICEF in the district of Foumbouni [29]. A tripartite group (PNUD, WHO and the Comorian government) organized the evaluation and adjustment of the programme.

However, chloroquine resistance spread over the country and compromised the campaigns. This resistance was first detected in 1988 by an in vivo study [33]. By the beginning of 1991, the rate of in vivo RII/RIII resistance increased to 22.2% in Anjouan [33, 34]. This led to the introduction of a sulfadoxine–pyrimethamine intermittent preventative treatment for pregnant women [38]. Studies conducted in 2001 revealed parasitological failure rates between 31.2 and 73.1% and overall failure rates (clinic and parasitological) between 50 and 88% for a 14-day follow-up protocol [33]. The government decided to change the national policy to artemisinin-based combination therapy (ACT) to follow WHO recommendations. After evaluation, artemether–lumefantrine was chosen in 2005 [41–43]. In 2006, malaria was still responsible for more than 38% of outpatient consultations and 60% of hospitalizations [42, 43].

### Mayotte and Réunion

In 1869, the introduction of malaria occurred suddenly in Réunion 3 years after it appeared in Mauritius. Early in the 20th century, the first measures of control included the control of wetlands and damps areas, and the use of petrol to destroy larvae. However, these actions were usually applied locally with poor effects on the overall epidemic. In 1914, an office was created to coordinate prophylaxis and disinfection, and in 1949, the first control programme for malaria was established using DDT [44]. After preliminary studies in Mauritius [45] and in Réunion in 1948 [46], in-house spraying (IHS) was organized between 1945 and 1952 [45, 47, 48] in Réunion, followed by partial spraying until 1962. These actions were very efficient, and in 1973, the interruption of transmission was declared [14]. Since 1973, the strategy of malaria control has been based on the passive detection of cases, the control of imported cases, and the destruction of larvae [47, 49]. In Mayotte, the control of malaria followed the same process. Before 2001, the strategy was based on vector control, the systematic treatment of clinically suspected malaria cases, and chemo-prophylaxis for pregnant women [50]. Since 2001, rapid diagnostic tests are systematically used to confirm the diagnosis.

### Malaria cases exported from Comoros to other countries

Due to the economic situation of the country, many Comorian people migrated abroad from their country during the past 40 years. In France, the Comorian population is estimated at 200,000 with 40,000 Comorian inhabitants in Réunion. Marseille (France) hosts more than 50,000 Comorians and is thus the largest ‘Comorian town’ in the world [51]. These expatriates used to return to the archipelagos, either during school/university holidays or for local festivals, particularly during the traditional “great weddings” period from July to September (a Great wedding is a traditional wedding separate from administrative and religious weddings and is organized by middle-aged people who want to obtain an honorific status in the community). These people are often infected by malaria during these periods and return to Europe with parasites. They represent the major source of imported cases of malaria in Marseilles [52, 53]. The French National Reference Centre for Malaria located in Marseilles collects sufficient data to be considered a reference centre for Comoros as well [54, 55]. However, due to the proximity of Mayotte (and its international airport), the population from Great Comoro travels more than the populations from the other islands, and most of the Comorians in Marseilles originate from this island. Indeed, imported cases in Marseille represent mostly the parasite populations of the Great Comoro, which differ from those from Moheli, Anjouan and Mayotte. Previous studies conducted in Marseilles showed that these parasites presented a higher frequency of mutations associated with drug resistance than isolates collected locally. Genetic analysis confirmed the proximity between malaria isolates imported in Marseilles and those from Great Comoro [56, 57]. The extrapolation of data obtained on malaria in Marseilles to the entire archipelago could thus be hazardous, particularly concerning drug resistance.

## Current strategies (2008–2015)

### Vector control strategies

After many years of studies, the government decided to strengthen malaria control and to guarantee universal access to interventions through a national plan. This plan was based on an extended distribution of long-lasting insecticide impregnated bed nets (LLINs) and on the implementation of indoor residual spraying (IRS). These actions began in Moheli in 2008–2010, and 340,000 bed nets were distributed, reaching a theoretical 92% coverage of the all households and a 60% rate of use. Larvivorous fishes were introduced in the cisterns in many places in the Great Comoro and in some localities in Anjouan and Moheli. The IRS project began after preliminary studies on the sensitivity of the mosquitoes to insecticides and based on the cartography of the sites to plan resources.

### A controversial tentative control method: mass distribution of anti-malarials (MDA)

By the end of 2007, a new programme was proposed on the island of Moheli (Fig. 2), conducted by an academic/industry Chinese consortium. Moheli was chosen because of its small population and the low transmission rate of malaria. A treatment of the entire population based on artemisinin–piperaquine (Artequick, Artepharm Co., China) was proposed, followed by primaquine alone, without any association with vector control. This strategy and this product are controversial because they were not previously fully evaluated in terms of efficacy or of acceptance by the population. Without clear evaluation of the programme, this action was resumed in Anjouan at the end of 2012. More than 80% of the people from Moheli and Anjouan received a curative dose of ACT (artemisinin–piperaquine, Artequick). A spectacular positive result was obtained thus far with more than a 98% decrease in the prevalence of malaria in Moheli and in Ndzouani. This result convinced the Comorian authorities to extend the experimentation to the island of Ngazidza in October 2015. This one-shot strategy gave much less convincing results due to the larger population and higher rate of transmission. Indeed, the proportion of patients attending consultations for malaria decreased slowly after these actions from 50% in 2004 to 36% in 2010. Only 53 deaths were registered in 2010. Therefore, in the population, the mortality of children under 5 years decreased sharply from 3.75‰ in 2005 to 0.75‰ in 2010. In the same year, the asymptomatic carriage of parasites decreased to 10.6% in children in Great Comoro, 9.1% in Anjouan and 5.4% in Moheli. A total of 103,670 malaria cases (mild and severe) were reported by public and private health centres in children younger than 5 years old in Anjouan and Great Comoro. In 2011, preliminary results from parasitological surveys showed a 6.3% asymptomatic carriage rate of parasites in the town of Fomoboni and 1.7% in the sanitarian district of Wanani [38], with an overall prevalence of 8.9%. In 2016, the threshold of pre-elimination was reached [35]. A total complete lack of information distributed to the public induced a context of suspicion among the population. No long-term evaluation of the efficacy was planned.

## Conclusions

### An advocacy for the sustainability of control strategies

Malaria transmission appeared in the Comoros Islands late during the 19th century as a result of the intensification of human migrations. The first set of actions developed against malaria consisted of multiple small, short-term actions that were not coordinated. They induced moderate, short-term modulation of the transmission. A second period occurred with more coordinated actions, such as the distribution of LLINs and training of health workers. Control of transmission was obtained, but without implementation of periodic evaluations. Consequently, the situation reverted to the previous level of transmission. After one century of focalized actions, the Health Ministry attempted to globally control the transmission in the archipelagos by implementing (i) training of health workers, (ii) management of cases and (iii) mosquito control. To date, the major strategy implemented is MDA, which seemed efficient only in two of the three islands. The transmission on the different islands reached different levels: (i) a pre-elimination state in Anjouan and Moheli, (ii) and a control step in the Great Comoro. Following this success, the Comorian government advocated for a new programme targeting elimination by 2020, with no autochthonous cases by the end of 2017. However, several questions remain for the future.

To date, the MDA strategy implemented by private companies is equivalent to the strategy deployed during the 1950s with chloroquine in other African countries. It appears efficient in decreasing transmission in some islands of the archipelago, such as Moheli. In the Great Comoro, the efficacy was much lower due to a higher rate of transmission. This programme is conducted by a private–public Chinese consortium with little involvement of the local teams and poor information provided to the population. It is not coordinated with other actions. The product used is only in phase 4 evaluation, and the total cost for these actions is supported by the Chinese government with poor transparency to the public. Thus, it cannot truly be considered a “national strategy”. Moreover, its sustainability can be questioned. No long-term evaluation of the efficacy of the programme was performed, and the cost to maintain this action has not been clearly calculated. The recontamination of the biotope can also be a long-term pitfall due to the huge level of uncontrolled exchange between the Comoros Islands and their neighbouring countries. The introduction of drug-resistant isolates can also occur with travellers from India, South East Asia or East Africa. These commercial links are poorly controlled, and no survey of the travellers has been organized. The risk of introducing artemisinin-resistant strains is thus quite high. Recontamination of the biotope and the introduction of drug-resistant strains are the two major pitfalls compromising the efficacy of MDA. In the context of Comoros, the major risk is linked to the introduction of resistant strains, rather than local emergence. The Great Comoro Island, which is still experiencing continuous transmission, can easily be a source of recontamination of the other islands. In addition, insecticide resistance of mosquitoes, which is usually associated to treatment of agricultural fields with pesticides, needs to be investigated regularly.

The cost of the interventions is another major pitfall of the strategy. Aside from the cost of the MDA itself (which is unknown), the prevention of recolonization of the different islands by parasites will also have a huge cost. The islands of Mauritius and Réunion can illustrate these difficulties because they are free of malaria but not of Anopheles. The vector control programme currently costs more than 10 million euros annually in Réunion, whereas in Mauritius, the control of travellers is based on a tough organization. The capacity of the Comoros government to support this cost can be questioned. The global cost to reach elimination can be in the same range or higher than that for control.

### What can be proposed for the future?

The first recommendation is to establish a national survey strategy, which has been lacking for decades. Only cross-sectional studies have been conducted, and mainly by foreign teams. Dozens of actions have been performed over short periods without evaluating the results. To move forward to the elimination step, laboratory facilities are necessary for both the diagnosis of malaria cases and for the evaluation of the actions and detection of subclinical cases. PCR methods for the detection of very low parasite densities and more immunological methods for the retrospective detection of contact with the parasite must be developed in local laboratories. Using these approaches, it will be possible to survey the overall transmission. Detecting antibodies against parasites in children can be used to evaluate the local recrudescence of transmission. Assessing the drug sensitivity of the parasites and of the vectors is also important in a context of low transmission associated with MDA. Stakeholders must first support the development of laboratory facilities under the supervision of the NMCP.

The second recommendation is to enhance the training of health workers and improve the information provided to the population. The MDA programme is the last of a long list of projects conducted without the clear participation of the population and of local teams. Stakeholders should support the training of local malariologists to conduct the national programme and of health workers, who can work more closely with the inhabitants. They will be able to locally adapt the “classical” actions proposed by the NMCP and conduct surveys on the efficacy or failure of the treatment.

Overall, the last point is to secure financial support, which is not obvious in a context of pre-elimination. When the number of human cases decreases and resources are limited, governments can rapidly re-attribute funds for the control of more prevalent diseases, which will compromise the sustainability of the programme.

## References

[1] Ouledi A, Toilibou A. Lutte contre les vecteurs du paludisme. Moroni: PNLP-Comores; 2012. A5.4.

[2] Mouchet J, Carnevale P, Coosemans M, Julvez J, Manguin S, Richard-Lenoble D (2004). Biodiversité du paludisme dans le monde.

[3] Blanchy S, Benthein F, Sabatinelli G (1987). Epidémiologie du paludisme en République fédérale Islamique des Comores. Cahiers ORSTOM. Série Entomologie Médicale et Parasitologie.

[4] Tchen J, Ouled A, Lepére JF, Frerrandiz D, Yvin JL (2006). Epidémiologie et prévention du paludisme dans les îles du sud-ouest de l’Océan Indien. Med Trop.

[5] Brunhes J (1975). Les moustiques de l’archipel des Comores. Cahiers ORSTOM. Série Entomologie Médicale et Parasitologie.

[6] Julvez J, Blanchy S (1988). Paludisme dans les îles de l’archipel des Comores, Aspects historiques et géophysiques. Considérations épidémiologiques. Bull Soc Pathol Exot.

[7] Le Goff G, Goodman SM, Elguero E, Robert V (2014). Survey of the mosquitoes of Mayotte. PLoS ONE.

[8] Damir BA. Vers l’élimination du paludisme dans l’archipel des Comores, No. 16–17. Paris: Yamkobe Paris CNDRS, KomEdit. 2008.

[9] Anonimous. Programme Nationale de Lutte contre le Paludisme des Comores. Direction Nationale de la Santé (ed.). 2012.

[10] Julvez J (1995). Historique du paludisme insulaire dans l’Océan Indien (partie sud-ouest). Cahiers Santé.

[11] Bernardin de St Pierre JH. Voyage à l’île de France, Paris, Mequignon-Marvis, 1818 (réédition) ed. Océan Indien, Maurice, 1986.

[12] De Flacourt E (1861). Histoire de la grande île Madagascar.

[13] Lougnon A. Sous le signe de la tortue: voyages anciens à l’île Bourbon (1611–1725). 3éd. Saint Denis; 1970.

[14] Julvez J, Mouchet J, Ragavood C (1990). Epidémiologie historique du paludisme dans l’archipel des Mascareignes (Océan Indien). Ann Soc Belge Méd Trop.

[15] Gevrey A (1870). Les Comores.

[16] Mohamed EH (1995). Paludisme à Mayotte: passé, présent, futur. Cahier Santé.

[17] Ouledi A (1995). Epidémiologie et contrôle du paludisme en République fédérale islamique de Comores. Cahiers Santé.

[18] Lafont M (1901). L’île d’Anjouan. Annales d’Hygiène et de Médecine Coloniales.

[19] Lafont M (1905). Mohéli. Annales d’Hygiène et de Médecine Coloniales.

[20] Raynal J (1928). Enquête sanitaire à la Grande Comore en 1925: observation de paludisme à forme épidémique. Bull Soc Path Exot.

[21] Brunhes J (1975). Les moustiques de l’archipel des Comores 1-Inventaire, répartition et description de quatre espèces ou sous-espèces nouvelles. Cahiers ORSTOM. Série Entomologie Médicale et Parasitologie.

[22] Courjault F. Recherche sur les origines du paludisme et sur les médications utilisées dans l’archipel des Mascareignes. Université de Tours, Thèse Pharmacie No. 5, 1983.

[23] Brygoo ER, Escolivet J (1955). Enquête sur la filariose aux Comores, à Mayotte et à Mohéli. Bull Soc Path Exot.

[24] Grejbine A. Culicidae Anophelinae. In: Faune de Madagascar, Tome XXII ORSTOM-CNRS Paris, vol. 36. 1966. p. 1–6.

[25] Subra R, Hebrard G. Étude écologique des moustiques de Mayotte vecteurs du paludisme et de la filariose de Bancroft en vue de leur contrôle. Service de Santé de Base et des Grandes Endémies, Moroni. Rapport ORSTOM 1974.

[26] Petrarca V, Sabatinelli G, Di Deco MA, Papakay M (1990). The *Anopheles gambiae* complex in the Federal Islamic Republic of Comoros (Indian Ocean): some cytogenetic and biometric data. Parassitologia.

[27] Ayala D, Goff GL, Robert V, De Jong P, Takken W (2006). Population structure of the malaria vector *Anopheles funestus* (Diptera: Culicidae) in Madagascar and Comoros. Acta Trop.

[28] Mouchet J, Robert V, Carnevale P, Ranvaonjanahary C, Coosemans M (1991). Le défi de la lutte contre le paludisme en Afrique tropicale : place et limite de la lutte antivectorielle. Cahiers Santé.

[29] Programme National de Lutte contre le Paludisme des Comores. Rapport d’Activité 2006. Direction Nationale de la Santé. 2007.

[30] Sabatinelli G, Majori G, Blanchi S, Fayaerts P, Papakay M (1990). Expérimentation du poisson larvivore *Poecilia reticulata* dans la lutte contre le paludisme en RFI des Comores. Rapport d’activité.

[31] Julvez J. Anthropisation et paludisme, Eco-épidémiologie historique du paludisme dans les archipels du sud-ouest de l’Océan Indien: Comores, Seychelles et Mascareignes. Thèse Sciences, Toulouse. 1993.

[32] Blanchy S, Benteinf S, Sabatinelli G (1987). Epidémiologie du paludisme en République Fédérale Islamique des Comores: Données actuelles. Cahiers ORSTOM. Série Entomologie Médicale et Parasitologie.

[33] Le Bras J, Simon F, Ramanamirija JA, Calmel MB, Hatin I (1987). Sensibilité de *Plasmodium falciparum* aux quinoléines et stratégies thérapeutiques : comparaison de la situation en Afrique et à Madagascar entre 1983 et 1986. Bull Soc Pathol Exot.

[34] Feillet N, Agnamey P, Druilhe P (1993). Résistance in vivo à la chloroquine de *Plasmodium falciparum* à Anjouan (Comores). Bull Soc Pathol Exot.

[35] Randrianarivelojosia M, Sajondra-Harisoa JL, Raharimalala L (2003). Evaluation in vitro de la sensibilité de *Plasmodium falciparum* à la chloroquine dans la région de l’Océan Indien dans le cadre du réseau d’étude de la resistance (RERE). Cahiers Santé.

[36] Ouledi A, Toyyb M, Aubry P, Gauzere BA (2012). Histoire sanitaire et enjeux sanitaires de l’Union des Comores en 2012. Médecine et Santé Tropicale.

[37] PNLP-Comores (2009). Plan stratégique national de lutte contre le paludisme 2007–2014.

[38] PNLP Comores (2014). Plan stratégique national de lutte contre le paludisme 2012–2016.

[39] Blanchy S, Julvez J, Mouchet J (1999). Stratification épidémiologique du paludisme dans l’archipel des Comores. Bull Soc Pathol Exot.

[40] Sabatinelli G, Majori G, Blanchy S, Fayaerts PH, Papakay M. Expérimentation du poisson larvivore *Poecilia reticulata* dans la lutte contre le paludisme en RFI des Comores. WHO report. 1990. No 1060.

[41] Blanchy S, Benthein F (1989). Chimiosensibilité in vivo de *Plasmodium falciparum* en République Fédérale Islamique des Comores. Bull Soc Pathol Exot.

[42] Programme National de Lutte contre le Paludisme des (2011). Comores: Revue de la performance du programme paludisme Moroni (Comores).

[43] Silai M, Moussa M, Abdalli Mari M, Astafieva-Djaza M, Hafidhou A, Oumadi M (2007). Surveillance de la chimiosensibilité du paludisme à *Plasmodium falciparum* et changement de politique dans l’Union des Comores. Bull Soc Pathol Exot.

[44] WHO. Elimination du paludisme sur l’île de la Réunion: 40 ans plus tard. Bibliothèque de l’OMS. 2014. ISNBN 9789242507737.

[45] Dowling MAC (1951). An experiment in the eradication of malaria in Mauritius. Bull World Health Organ.

[46] Dufour G, Hamon J (1951). Rapport sur la lutte antipaludique; campagne 1950–1951.

[47] Hamon J, Dufour G (1954). La lutte antipaludique à la Réunion. Bull World Health Organ.

[48] Dufour G, Hamon J (1952). Rapport sur la lutte antipaludique; campagne 1951–1952.

[49] Bruce-Chwatt LJ, Draper CC, Konfortion P (1973). Seroepidemiological evidence of eradication of malaria from Mauritius. Lancet.

[50] Gatier J, Blanchy S (1982). Le paludisme à Mayotte et son évolution du 1976 à 1980. Cahiers ORSTOM.

[51] Ministère Français des Affaires Etrangères. Présentation de l’Union des Comores. 2008. http://www.diplomatie.gouv.fr/fr/dossiers-pays/comores/presentation-de-l-union-des-comores/. Accessed 22 Sept 2017.

[52] Parola P, Minodier P, Soula G, Jaffre Y, Badiaga S, Retornaz K (2005). Le paludisme d’importation à l’Hôpital-Nord de Marseille en 2001–2003: étude prospective de 352 cas. Med Mal Infect.

[53] Mohamed A. Etude descriptive rétrospective de 823 cas de paludisme importés des Comores à Marseille entre 2001 et 2005. Thèse de la Faculté de Médecine de Marseille. Marseille: Université de la Méditerranée. 2007.

[54] Parola P, Gazin P, Pradines B, Parzy D, Delmont J, Brouqui P (2004). Marseille: a surveillance site for malaria from the Comoros Islands. J Travel Med.

[55] Parola P, Pradines B, Simon F, Carlotti MP, Minodier P, Ranjeva MP (2007). Antimalarial drug susceptibility and point mutations associated with drug resistance in 248 *Plasmodium falciparum* isolates imported from Comoros to Marseille, France in 2004–2006. Am J Trop Med Hyg.

[56] Anderson TJ, Haubold B, Williams JT, Estrada-Franco JG, Richardson L, Mollinedo R (2000). Microsatellite markers reveal a spectrum of population structures in the malaria parasite *Plasmodium falciparum*. Mol Biol Evol.

[57] Bogreau H, Renaud F, Bouchiba H, Durand P, Assi SB, Henry MC (2006). Genetic diversity and structure of African *Plasmodium falciparum* populations in urban and rural areas. Am J Trop Med Hyg.

